# Microgravity activates monocyte ERK1/2 signaling and modulates the response to lipopolysaccharide

**DOI:** 10.1186/s10020-025-01407-y

**Published:** 2025-11-29

**Authors:** Ruslan A. Mammadov, Melle P. C. van Hulten, Max K. Bakker, Auke P. Verhaar, Maikel P. Peppelenbosch

**Affiliations:** 1 Department of Gastroenterology and Hepatology, Erasmus University Medical Center Rotterdam, Rotterdam, The Netherlands; 2 Department of Surgery, Erasmus MC Transplant Institute, University Medical Center Rotterdam, Rotterdam, The Netherlands

**Keywords:** Microgravity, ERK1/2, LPS, 1G, ΜG, Monocytes, Single-Cell analysis, Fluorescent microscopy

## Abstract

**Background:**

Microgravity alters immune cell function, potentially compromising host defense during spaceflight. Because appropriate immune regulation is also critical in chronic inflammatory and autoimmune conditions, insights from spaceflight biology may have broader implications for human health. Monocyte activation via the p44/42 MAPK pathway is central to inflammatory responses, yet the influence of microgravity on this signaling cascade remains incompletely understood. This study aimed to determine how microgravity affects basal and lipopolysaccharide (LPS)-stimulated ERK1/2 kinases (also known as p44/42 MAP kinases) activity in human monocytes, focusing on signaling state redistribution at both single-cell and population levels.

**Methods:**

Monocytes were cultured during spaceflight under either normal gravity (1G) or microgravity (µG) and exposed to LPS or control conditions. MAPK activity was quantified and analysed to assess basal signaling, stimulus responsiveness, and variability within the population.

**Results:**

Basal MAPK activity was significantly elevated in µG compared with 1G monocytes (*p* = 0.0181). LPS stimulation robustly increased MAPK activity in 1G cells (*p* = 0.0267) but not in µG (*p* = 0.6752). Although baseline signaling was higher in µG, LPS responses in µG and 1G were not significantly different (*p* = 0.7905). Under microgravity, the cell population displayed broader signaling distribution and a larger non-responsive fraction. Although baseline signaling was higher in µG net LPS responsiveness was diminished compared with 1G.

**Conclusion:**

Microgravity redistributes monocyte signaling states, increasing basal ERK1/2 activity while attenuating rapid stimulus-induced activation and expanding the non-responsive cell fraction. These findings provide new mechanistic insight into how microgravity shapes immune signaling and highlight cellular heterogenety as a critical determinant of immune regulation during spaceflight.

**Supplementary Information:**

The online version contains supplementary material available at 10.1186/s10020-025-01407-y.

## Background

Autoimmune diseases remain one of the most complex challenges in modern medicine, affecting hundreds of millions worldwide (Abend et al. 2024; Khandwala et al. 2025). They are characterized by the immune system attacking the body’s own tissues, leading to chronic inflammation, progressive organ damage, and impaired function (Engel et al. 2020; Hidaka and Amino 1999). Beyond individual suffering, these diseases contribute significantly to morbidity, mortality, and healthcare costs (Engel et al. 2020; Schroeder et al. 2019).

Therapeutic options remain limited. Current treatments primarily involve immunosuppressive drugs such as glucocorticoids, calcineurin inhibitors, and biologics targeting specific cytokines or lymphocyte subpopulations (Hidaka and Amino 1999; Efferth and Oesch 2021; Kanatoula et al. 2024; Sambataro et al. 2025; Yan et al. 2025). While effective at suppressing pathological immune activity, these treatments have significant drawbacks. Long-term high-dose use causes systemic toxicity, severe side effects, and increased susceptibility to infections and malignancies (Mihatsch et al. 1990; Meyer et al. 2010; Dan et al. 2014; Nakajima et al. 2009). This underscores the need for new strategies that selectively modulate immune responses without compromising overall immunity.

An underexplored model of immune regulation is the impact of microgravity on astronauts’ immune systems during and after spaceflight (Stowe et al. 1999, 2001; Kaur et al. 2005). Microgravity causes pronounced immune dysfunction, including impaired T- and B-cell activation, altered cytokine production, and disrupted intracellular signaling (Kaur et al. 2005; Boonyaratanakornkit et al. 2005; Grosse et al. 2012; Nichols et al. 2006). Unlike pharmacological immunosuppression, these effects arise from environmental factors without systemic toxicity, making microgravity a unique natural model for studying immune regulation and identifying therapeutic targets (Okamura et al. 2024; Loon et al. 2020; Isasi et al. 2022; Elsaesser et al. 2023).

At the molecular level, microgravity affects many aspects of immune function. T cells exhibit reduced proliferation and altered signaling responses, B cells produce fewer antibodies, and innate immune cells show impaired phagocytosis and mediator secretion (Kaur et al. 2005, 2008; Boonyaratanakornkit et al. 2005; Grosse et al. 2012; Davis et al. 2024; Groot et al. 1990). Early studies generally reported immune suppression, but mechanisms remain unclear (Williams et al. 2009; Hicks et al. 2023). Most analyses were population-level, masking intra-population heterogeneity (Khandwala et al. 2025; Schroeder et al. 2019; Lasyte et al. 2025; Wen and Li 2023). Cellular heterogeneity is now recognized as crucial for coordinating effective immune responses (Yan et al. 2025; Alvarez and Rojas 2023; Coaccioli et al. 2005; Li et al. 2025; Ma et al. 2019; Qiu et al. 2024; Zhang et al. 2021).

The mitogen-activated protein kinase (MAPK) cascade is central to immune regulation (Li et al. 2017; Huang et al. 2009; Kasper et al. 2010; Ng et al. 2024). It integrates extracellular stimuli, including cytokines and stress signals, into intracellular responses, controlling gene expression, survival, activation, and differentiation (O’Sullivan et al. 2009; Schuler et al. 2024). In immune cells, ERK1/2 kinases (also known as p44/42 MAP kinases) regulate the transcription factors c-Fos and c-Jun, which form the AP-1 complex, a key regulator of cytokine production, lymphocyte activation, and inflammation (Trelease et al. 1999; Torres et al. 2013; Monje et al. 2005). Dysregulation is linked to both autoimmune hyperactivation and impaired pathogen defense.

Microgravity reduces c-Fos and c-Jun activity, suggesting that disrupted MAPK signaling may underlie observed immune changes (Nichols et al. 2006; Groot et al. 1990; Verhaar et al. 2014).

Cellular heterogeneity is particularly relevant for monocytes, which vary in functional states and activation programs (Ma et al. 2019; Mussbacher et al. 2023; Geissmann et al. 2010). Hyperactive monocyte subpopulations drive inflammation in autoimmune diseases, while other cells may remain inactive (Ma et al. 2019; Schildberger et al. 2013). Population-level analyses obscure these differences, necessitating single-cell techniques like flow cytometry, quantitative signaling measurements, and imaging (Zhou et al. 2012) .

Monocytes are key innate immune responders, producing pro-inflammatory cytokines and initiating adaptive immunity (Ma et al. 2019). In autoimmune diseases, hyperactivated monocytes cause tissue damage via excessive mediator production and recruitment of other immune cells (Li et al. 2025; Mussbacher et al. 2023). Their activity is tightly regulated by MAPK signaling and AP-1, making them an ideal model for studying microgravity’s effects on intracellular signaling.

Microgravity triggers a complex reorganization of functional states rather than simple suppression (Verhaar et al. 2014). Some monocytes show elevated basal MAPK activity but reduced stimulus responsiveness, combining partial pre-activation with low inducibility. Cellular responses are heterogeneous, with some cells pre-activated and others functionally silent. Mechanistically, these effects may result from cytoskeletal disruption and impaired mechanotransduction, affecting receptor organization, signal propagation, and transcription (Lv et al. 2023; Ullrich et al. 2008; Wu et al. 2024).

Understanding microgravity-induced immune changes is critical for astronaut health during long-term missions, including future lunar and Martian flights (Hicks et al. 2023). Beyond space related research, microgravity offers a natural model for selectively modulating immune responses without systemic toxicity, providing insights for novel autoimmune therapies (Elsaesser et al. 2023; ElGindi et al. 2021).

Rapid activation of the ERK1/2 pathway is a hallmark of early immune signaling, occurring within minutes after receptor engagement. In monocytes, such short timescale ERK responses orchestrate the initial transcriptional and functional activation programs that drive downstream inflammation and adaptive immunity. Comparable mechanisms have been observed in T cells exposed to microgravity, where acute ERK pathway modulation contributes to impaired activation dinamics (Tauber et al. 2013). In innate immune cells, microgravity multiplied pathways over different timescales, from early, kinase activation to longer-term differentiation processes (An et al. 2024). Moreover data from long duration spaceflight studies indicate that ERK signaling plays a pivotal role in macrophage development and immune homeostasis (Shi et al. 2021). However, it fair to say that the effect of microgravity on both constitutive ERK 1/2 activation as well as induced ERK ½ activation remain only partly understood.

Prompted by the considerations mentioned above, this study aimed to characterize MAPK signaling in human monocytes under microgravity at both population and single-cell levels, focusing on basal activity, stimulus responsiveness, and cytoskeletal contributions. Integration of quantitative, single-cell, and imaging methods provided a comprehensive view of how microgravity reshapes monocyte functional states, offering insights for both spaceflight immunology and the development of targeted therapies for autoimmune diseases.

## Materials and methods

This study was conducted following Institutional Review Board approval from the Academic Medical Center, and informed consent was obtained in accordance with the Declaration of Helsinki. Blood samples were collected from young healthy volunteers, whose health status was verified by physical examination, into Vacutainer tubes containing EDTA-K3 (Becton-Dickinson, Breda, the Netherlands). Peripheral blood mononuclear cells (PBMCs) were isolated using Ficoll-Paque Plus density gradient centrifugation (Amersham Biosciences AB), followed by washing and resuspension in IMDM supplemented with 10% fetal bovine serum, penicillin, streptomycin, and amphotericin. Blood was drawn within 10 h prior to launch.

Monocytes were subsequently isolated by positive selection with CD14 + MicroBeads (Miltenyi Biotec), yielding > 95% purity and > 98% viability, and cultured in RPMI-1640 medium supplemented with 10% fetal bovine serum, 1% penicillin/streptomycin, and 2 mM L-glutamine at a final density of 1 × 10⁶ cells/mL.

**Fig. 1:**
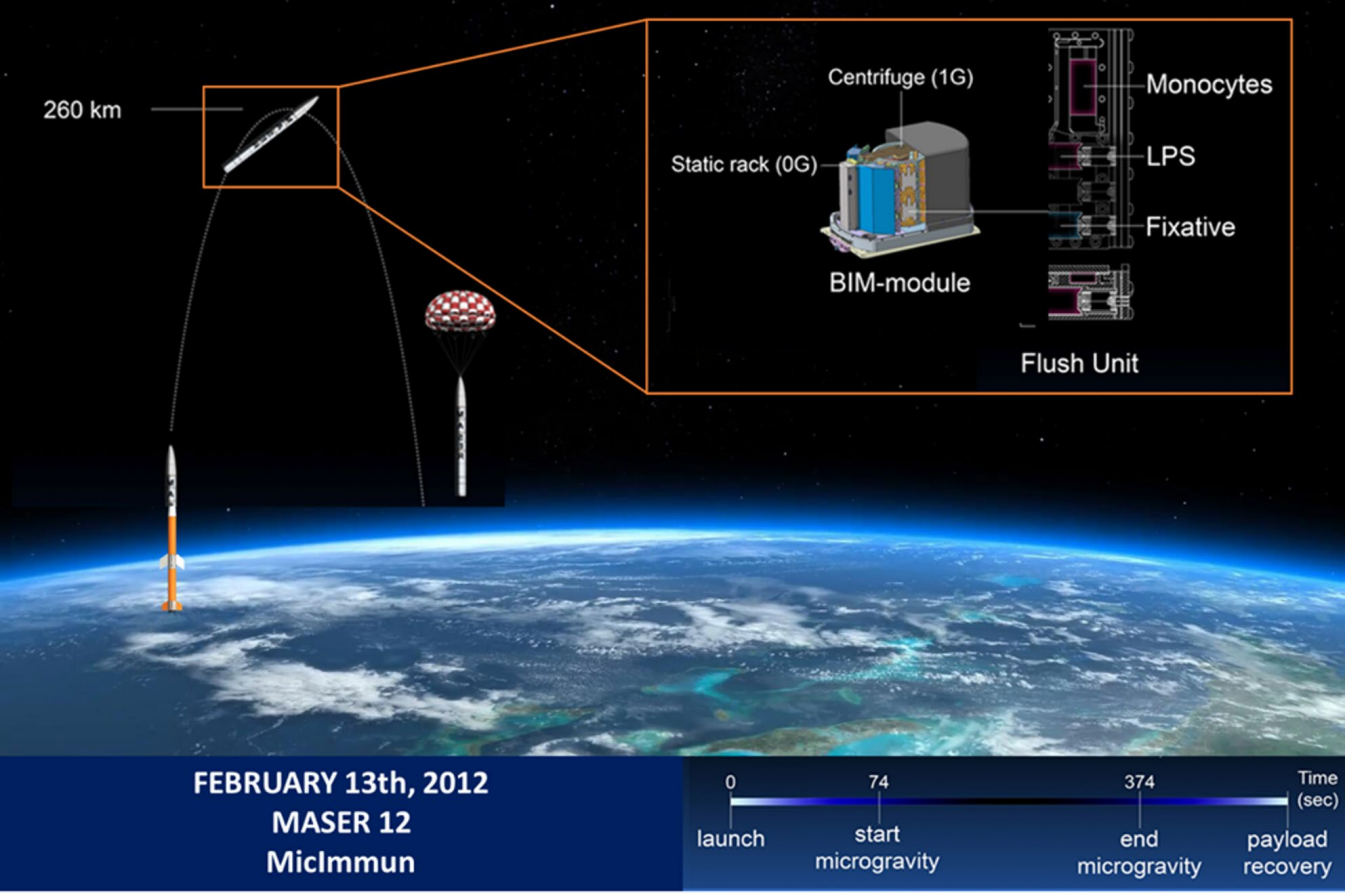
Overview of the experimental setup and timeline. The experiment was conducted in 2012 during the MASER-12 mission (MicroImmun). The mission utilized sounding rockets to carry the biological experiment into space. During the parabolic flight, the rocket reached an altitude of approximately 260 km, providing 5–6 min of microgravity. Experiments were performed in fully automated climate-controlled chambers called Flush Units (FUs), which were housed within the Biology in Microgravity (BIM) module. The BIM module regulated ambient temperature and supplied power to the FUs throughout the flight. As an onboard control, some FUs were mounted in a centrifuge that generated a 1 g force, while other FUs were placed on a static rack and exposed to microgravity. Prior to launch, monocytes were seeded onto two glass slides within a rectangular chamber inside each FU. At the onset of microgravity, a timed spring mechanism replaced the culture medium with medium containing lipopolysaccharide (LPS) to stimulate the cells. At the end of the microgravity phase, a second timed mechanism replaced the LPS-containing medium with fixative, terminating cellular activity

Experiments were performed aboard the MASER-12 sounding rocket (Zhang et al. 2021; Li et al. 2017), launched on 13 February 2012 from the Esrange Space Center (Swedish Lapland), which reached a maximum altitude of 260 km, providing approximately five minutes of microgravity (t = 74–374 s; <10⁻⁴ g/s) (Fig. 1).

Onboard 1G controls were maintained using a centrifuge integrated into the MASER-12 payload, which matched temperature, vibration, and acceleration profiles during flight. Ground controls with identical mechanical conditions were not feasible for this flight configuration; however, vibration tests and temperature controls on the BIM-2 module confirmed that mechanical forces did not influence cellular responses, ensuring comparability between 1G onboard and µG microgravity conditions (results not shown).

The payload returned to Earth by parachute. The experiments were carried out in the “Biology in Microgravity” (BIM) module developed by Dutch Space (Leiden, the Netherlands) and the Kubik module developed by ESA (Noordwijk, The Netherlands), loaded with automated experimental container units designed by the Centre for Concepts of Mechatronics (Nuenen, The Netherlands). These units included two experimental devices, each containing three chambers. The two outer chambers were connected via capillary tubes to the middle chamber, which contained the biological material. Special compartments were used for fixatives to prevent diffusion through rubbers and plastics. Electronically-controlled springs released the activation solution (LPS 100 ng/mL) and fixative (4% formaldehyde in PBS) at preset time points. LPS or vehicle control was administered shortly after the onset of microgravity, and fixative was applied exactly five minutes later. Technical challenges during flight and recovery limited the usable biological samples. Of 96 samples sent to space, only 31 from the MASER-12 flight qualified for further analysis. Blood samples were collected from healthy volunteers (Supplementary Table 1), whose health status was verified by physical examination.

The primary causes of sample loss included delayed or incomplete automated fixation in some experimental units, fluid transfer failure in the capillary system, and mechanical stress during launch and landing that compromised sample integrity. All retained samples were selected based on predefined quality control criteria, including cell viability, adequate fixation, and fluorescence staining quality. Microgravity and 1G control samples were handled identically during post-flight staining and processing to minimize potential bias introduced by manual handling. Although some variability may have influenced cell clustering patterns, these effects were equally distributed across experimental conditions. Payload and centrifuge accelerations were continuously monitored during flight, and vibration tests with the BIM-2 module confirmed that mechanical forces did not affect experimental outcomes.

Cells were maintained at 36.5 ± 0.5 °C throughout the experiments. Experimental conditions included four groups: µG with LPS, µG without LPS, 1G with LPS, and 1G without LPS, with three Flush Units assigned to each condition. Stimulations were performed within ten hours after blood collection. Following recovery, cells were washed with PBS and permeabilized with 0.1% Triton X-100 in PBS. Samples were incubated overnight at 4 °C with monoclonal rabbit antibodies against phospho-ERK1/2 (Thr202/Tyr204) (Cell Signaling Technology), followed by incubation with anti-rabbit Alexa Fluor 488 secondary antibody (Invitrogen) for 1 h at room temperature and counterstaining with DAPI.

Confocal images were acquired using a Leica TCS SP2 system equipped with 405-nm UV, 488-nm argon, and 543-nm HeNe lasers (Leica, Mannheim, Germany) and processed with ImageJ software (National Institutes of Health, Bethesda, MD) for construction of total fluorescence signal and automated quantification of nuclear versus cytoplasmic localization. For single-cell analysis, monocytes were manually segmented using predefined intensity thresholds and size criteria to exclude debris or cell clusters unsuitable for quantification. Random sampling was applied using a predefined grid across the imaging field. Only cells meeting viability criteria (the presence of the DAPI and ERK signal) and staining quality (approved by two independent observers) criteria were included in analyses, ensuring consistency across all experimental conditions. In addition an attempt was made to assess viability of the cultures. It was assumed that any living cell should contain a certain pERK signal, whereas a dead cell should only display a DAPI signal. If the amount of apparent dead cells exceeded > 2%, a sample was rejected for further analysis.

Random sampling was applied by selecting cells on a predefined grid, excluding clusters unsuitable for analysis.

The mean cellular intensity (*I* cell*)* was calculated as the multiple background-corrected total intensity divided by the number of cells:

$$\:I\:\text{c}\text{e}\text{l}\text{l}=\frac{I\:\text{t}\text{o}\text{t}\text{a}\text{l}-\sum\left(Ibackground,i\cdot\:Ai\text{}\right)}{N\:\text{c}\text{e}\text{l}\text{l}\text{s}}$$

where:

- *I* total​ = total integrated intensity.
- *I background*,* i​* = mean background intensity for each region/cell *i*.
- *Ai​* = area of region/cell *i*.
- N = number of cells.

Statistical analysis was performed using GraphPad Prism 9 and Microsoft Excel. Standard deviations were calculated for each group, and comparisons were made using independent two-sided Student’s t-tests with Welch’s correction where appropriate. For the 1G + LPS group, a one-sided t-test was applied when a directional change was hypothesized. Violin plots were used to display per-cell intensity distributions, and *p* < 0.05 was considered statistically significant.

## Results

To investigate the impact of microgravity on monocyte immune function, we analyzed p44/42 MAPK activity in human monocytes under normal gravity (µg (microgravity) real microgravity (µG) and 1G (onboard centrifugal control), with or without LPS stimulation. MAPK signaling is central to monocyte activation and inflammatory responses, serving as a robust indicator of basal activity, stimulus responsiveness, and population heterogeneity (Huang et al. 2009; Schildberger et al. 2013; Cuadrado And Nebreda 2010). To comprehensively assess these effects, we combined global activity measurements, single-cell analyses, decile-ranked population heterogeneity assessments, and fluorescence microscopy.

A total of 1,234 monocytes were analyzed across all conditions, with 312–321 cells per group included after quality control.

### Basal and LPS-Induced MAPK activity

**Fig. 2:**
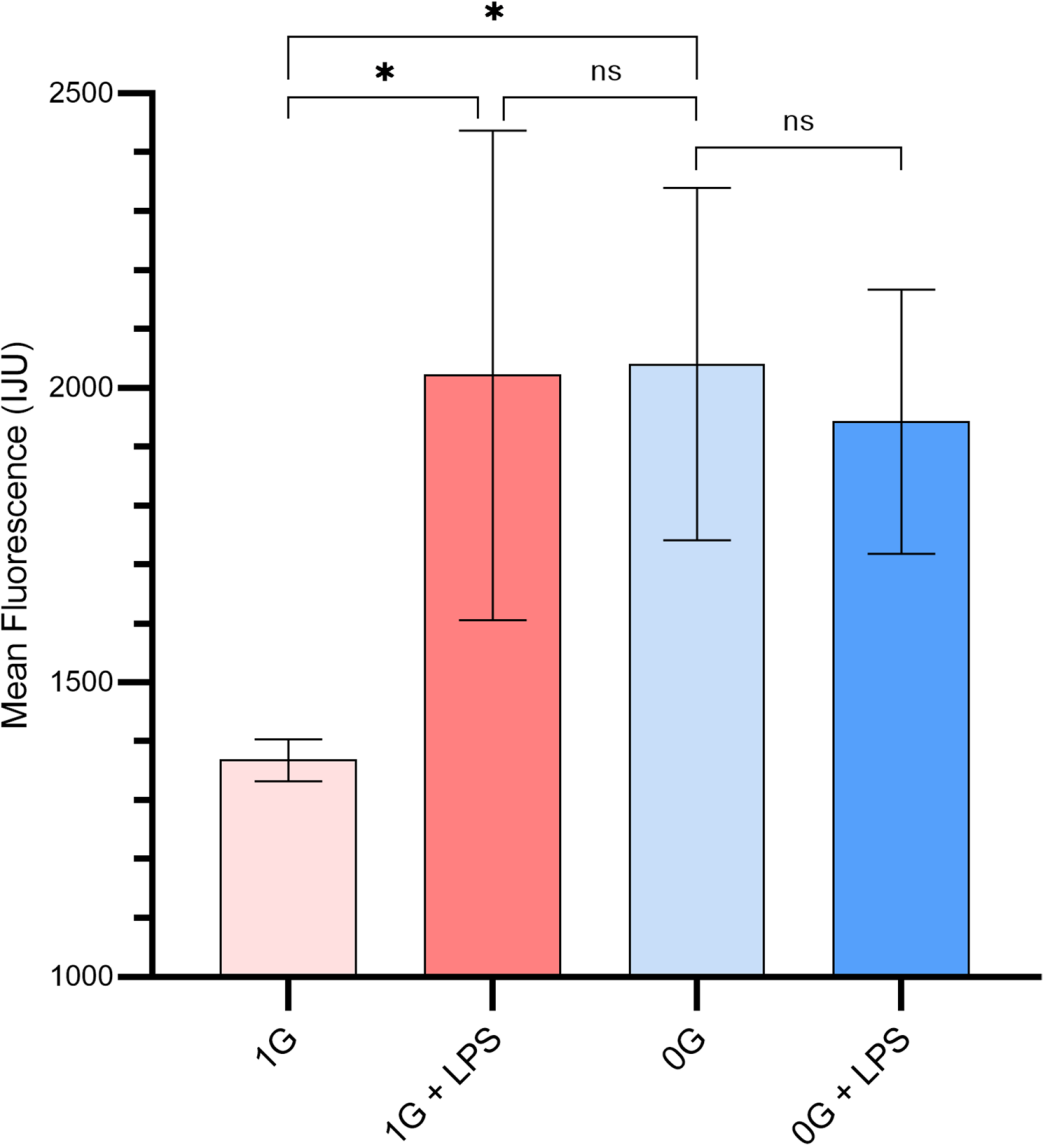
Global MAPK activity under normal and microgravity conditions. p44/p42 MAPK activity in monocytes under 1G and µG, with or without LPS. Bars indicate mean ± SD. LPS significantly increased activity in 1G, but not µG. Statistical comparisons: 1G vs. 1G + LPS, *p* = 0.0267; µG vs. µG + LPS, *p* = 0.6752; 1G vs. µG, *p* = 0.0181; µG + LPS vs. 1G + LPS, *p* = 0.7905

Global MAPK activity measurements revealed that basal MAPK levels were significantly higher in µG monocytes compared to 1G (*p* = 0.0181), while some cells under µG achieve activation levels comparable to 1G + LPS, the population as a whole exhibits a heterogeneous distribution of basal activity. Therefore, we refer to this state as ‘partially activated,’ reflecting that only a subset of cells is maximally active while others remain at lower basal levels (Fig. 2). LPS robustly increased MAPK activity in 1G monocytes (1G vs. 1G + LPS, *p* = 0.0267), confirming normal responsiveness. In contrast, µG monocytes did not exhibit a statistically significant increase upon LPS stimulation (µG vs. µG + LPS, *p* = 0.6752). This suggests that microgravity does not suppress MAPK activation per se but limits further inducibility, as a subset of cells may already be near maximal activation under basal µG conditions. Notably, standard deviations were higher in 1G + LPS, µG, and µG + LPS groups, reflecting heterogeneous activation within these populations.

These results suggest that microgravity simultaneously elevates basal signaling and reduces dynamic responsiveness, a pattern consistent with stress-induced pre-activation observed in other immune cell studies under microgravity conditions (Huang et al. 2009). The increased basal activity may represent compensatory signaling or a reprogrammed cellular state that limits further inducibility, which is critical for understanding impaired immune function in low-gravity environments.

### Single-cell distribution of MAPK activity

**Fig. 3:**
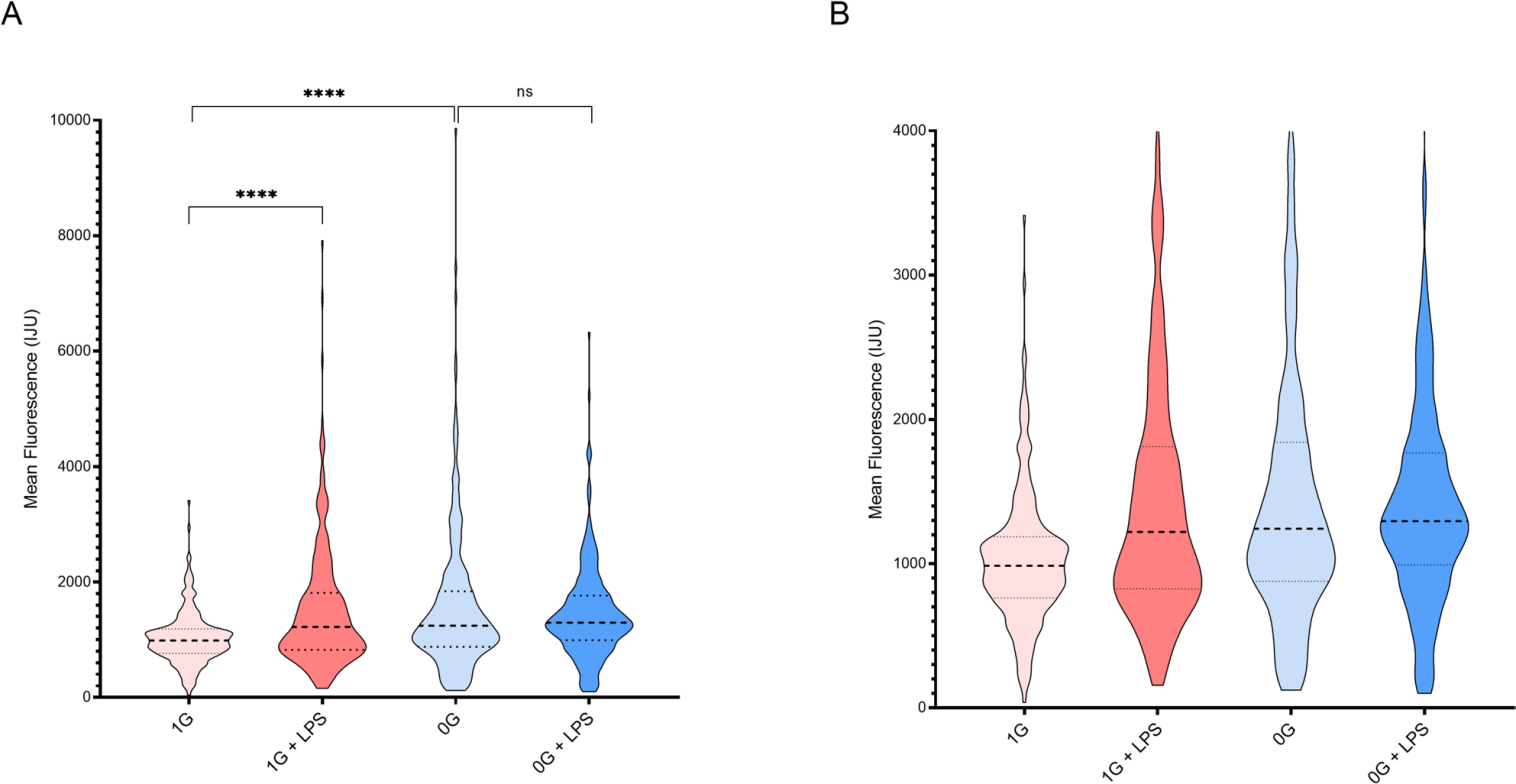
Single-cell MAPK activity distributions. Violin plots depict the distribution of p44/p42 MAPK activity in individual monocytes under each condition. Width reflects density of cells at each activity level. Outliers indicate highly activated cells, predominantly in µG, 1G + LPS, and µG + LPS groups. **A**) Violin plots showing the full single-cell data including outliers; **B**) Violin plots showing the same dataset with extreme outliers removed for visualization purposes, facilitating comparison of the central distribution. Statistical significance for violin plots was assessed using comparisons of mean and decile-ranked single-cell intensities with Student’s t-test and Welch’s correction where appropriate. *p* < 0.05 was considered significant

Violin plots of individual monocytes revealed pronounced heterogeneity in all experimental conditions (Fig. 3A and B). Under µG, the distributions were broader and skewed, with a larger fraction of cells showing low or absent pERK activation, reflecting increased functional heterogeneity and a higher proportion of non-responders. Outlier cells with high MAPK activity were most frequent in µG, but also observed in 1G + LPS and µG + LPS, indicating that subsets of cells can achieve maximal activation even under suppressed conditions. The 1G + LPS population exhibited a significantly broader distribution than 1G alone (*p* < 0.0001), as determined by comparisons of standard deviations and variance across single-cell measurements. This approach highlights increased heterogeneity, including a larger fraction of low- or non-responding cells under microgravity, beyond what is captured by mean intensity values alone. Quartile analyses confirmed minimal LPS effect in µG (*p* = 0.1755), while differences between 1G and µG were highly significant (*p* < 0.0001).

These single-cell observations reveal that microgravity does not uniformly suppress MAPK activity; instead, it reshapes the distribution, enhancing variability and creating a population in which only a fraction of cells is capable of strong activation. This heterogeneity has functional implications, as coordinated immune responses depend on synchronized signaling across the cell population.

### Population heterogeneity and ranked activity

**Fig. 4:**
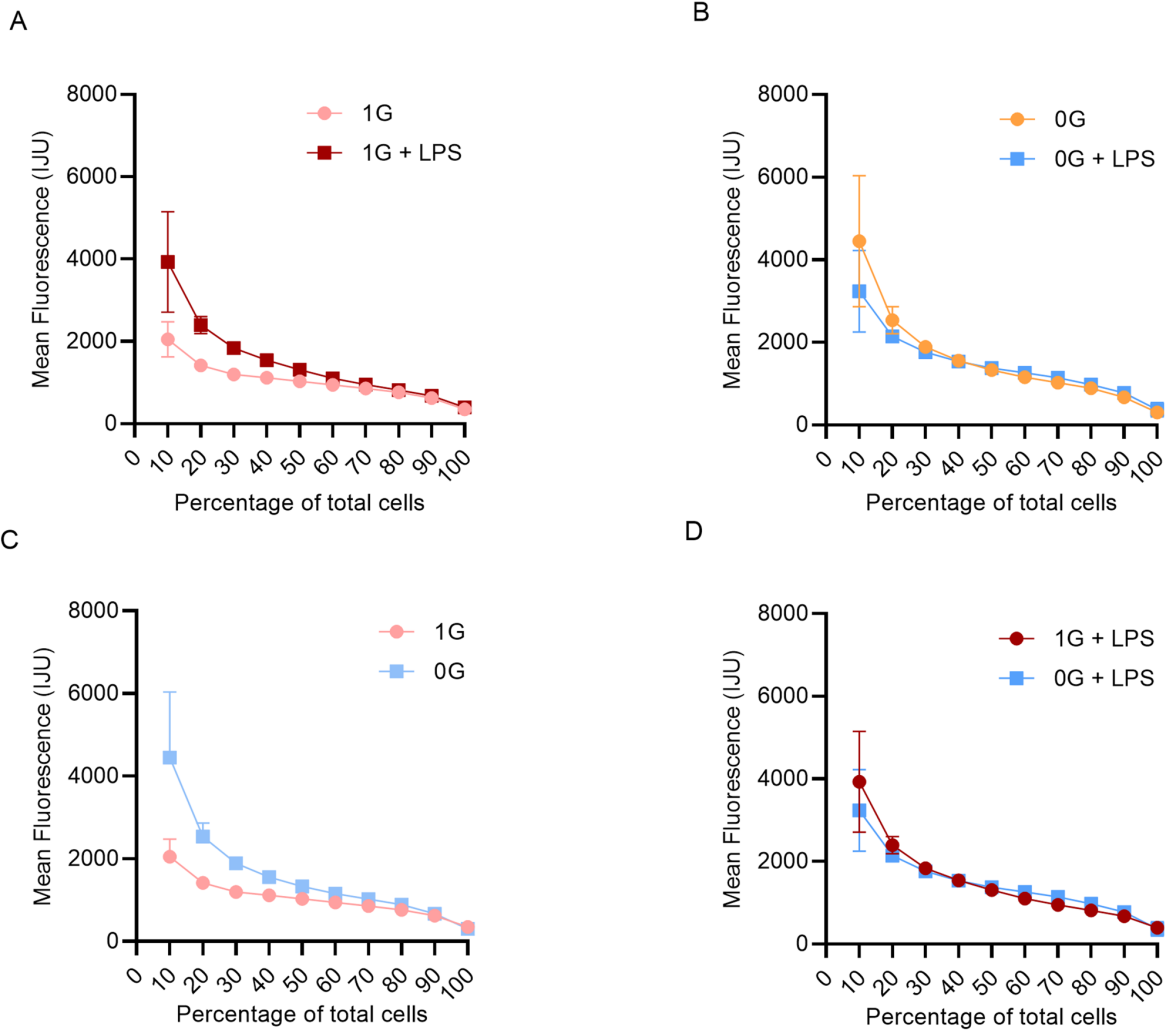
Population-level heterogeneity of MAPK activation. Monocyte activity ranked by deciles (10% increments). Mean activity is shown for each decile with SD. **A** 1G vs. 1G + LPS; **B** µG vs. µG + LPS; **C** 1G vs. µG; **D** 1G + LPS vs. µG + LPS. Reduced LPS responsiveness and increased variability are apparent under µG

To quantify heterogeneity of MAPK responses, monocyte activity was ranked by deciles (10% increments; Fig. 4A–D; Table 1). In 1G monocytes, LPS stimulation significantly increased MAPK activity across most deciles (10–90%). For example, at the 10th percentile, mean activity increased from 2048 in 1G to 3849 in 1G + LPS (Δ = 1801 ± 242.4, *p* < 0.0001), while at the 50th percentile, activity increased from 940.3 to 1100 (Δ = 160.0 ± 10.7, *p* < 0.0001). Significant induction was observed up to the 90th percentile (*p* = 0.0044), but not at the 100th percentile (*p* = 0.2009), suggesting that the most highly responsive cells were already maximally activated. These results confirm that in 1G, LPS robustly activates MAPK in the majority of the population, though responsiveness decreases toward the upper deciles.

**Table 1:** Summary of mean MAPK activity and statistical comparisons

Figure 4A. 1G vs. 1G + LPS						
Decile	*p* value	Summary	Significant?	Mean 1G	Mean 1G + LPS	Difference (± SEM)
10%	< 0.0001	****	Yes	2048	3849	1801 ± 242.4
20%	< 0.0001	****	Yes	1414	2394	980.8 ± 44.3
30%	< 0.0001	****	Yes	1194	1837	643.4 ± 23.8
40%	< 0.0001	****	Yes	1114	1544	430.0 ± 15.7
50%	< 0.0001	****	Yes	1030	1313	282.6 ± 12.5
60%	< 0.0001	****	Yes	940.3	1100	160.0 ± 10.7
70%	< 0.0001	****	Yes	858.0	950.5	92.5 ± 9.5
80%	< 0.0001	****	Yes	762.6	817.1	54.4 ± 9.9
90%	0.0044	**	Yes	625.7	675.4	49.7 ± 16.6
100%	0.2009	ns	No	348.1	396.8	48.7 ± 37.5

Figure 4B. µG vs. µG + LPS						
Decile	*p* value	Summary	Significant?	Mean µG	Mean µG + LPS	Difference (± SEM)
10%	0.0022	**	Yes	4447	3237	-1209 ± 371.4
20%	< 0.0001	****	Yes	2538	2141	-396.7 ± 72.0
30%	< 0.0001	****	Yes	1891	1762	-128.7 ± 21.3
40%	0.292	ns	No	1559	1538	-20.2 ± 18.9
50%	0.0017	**	Yes	1329	1374	45.6 ± 13.7
60%	< 0.0001	****	Yes	1159	1261	101.5 ± 11.8
70%	< 0.0001	****	Yes	1024	1142	118.9 ± 10.9
80%	< 0.0001	****	Yes	888.0	978.9	90.9 ± 8.6
90%	< 0.0001	****	Yes	669.0	775.3	106.3 ± 24.3
100%	0.1198	ns	No	304.0	368.0	64.0 ± 40.4

Figure 4C. 1G vs. µG						
Decile	*p* value	Summary	Significant?	Mean 1G	Mean µG	Difference (± SEM)
10%	< 0.0001	****	Yes	2048	4447	2399 ± 324.5
20%	< 0.0001	****	Yes	1414	2538	1124 ± 67.2
30%	< 0.0001	****	Yes	1194	1891	697.0 ± 25.2
40%	< 0.0001	****	Yes	1114	1559	444.5 ± 16.1
50%	< 0.0001	****	Yes	1030	1329	298.8 ± 11.5
60%	< 0.0001	****	Yes	940.3	1159	218.8 ± 11.5
70%	< 0.0001	****	Yes	858.0	1024	165.5 ± 88.4
80%	< 0.0001	****	Yes	762.6	888.0	125.4 ± 11.1
90%	0.0611	ns	No	625.7	669.0	43.3 ± 22.5
100%	0.2259	ns	No	348.1	304.0	-44.1 ± 35.9

Figure 4D. 1G + LPSvs µG + LPS						
Decile	*p* value	Summary	Significant?	Mean 1G + LPS	Mean µG + LPS	Difference (± SEM)
10%	0.0483	*	Yes	3849	3237	-611.7 ± 302.3
20%	0.006	**	Yes	2394	2141	-253.4 ± 51.3
30%	0.7577	ns	No	1837	1762	-75.1 ± 25.9
40%	0.0001	****	Yes	1544	1538	-5.8 ± 16.1
50%	< 0.0001	****	Yes	1313	1374	61.8 ± 14.6
60%	< 0.0001	****	Yes	1100	1261	160.4 ± 11.0
70%	< 0.0001	****	Yes	950.5	1142	191.9 ± 11.4
80%	< 0.0001	****	Yes	817.1	978.9	161.8 ± 15.7
90%	< 0.0001	****	Yes	675.4	775.3	99.9 ± 18.8
100%	0.4938	ns	No	396.8	368.0	-28.9 ± 41.8

Mean activity, standard deviation, and *p* values are presented for all group comparisons. The data confirm that LPS significantly activates MAPK in 1G monocytes but has minimal effect in µG. Microgravity increases population heterogeneity, reducing the overall responsiveness to LP; *p <0.05; **p <0.01; ****p <0.0001; *ns* not significant

In contrast, µG monocytes exhibited markedly reduced inducibility by LPS. While significant increases were detected at several lower and mid-deciles (e.g., 10%: Δ = − 1209 ± 371.4, *p* = 0.0022; 20%: Δ = − 396.7 ± 72.0, *p* < 0.0001; 60%: Δ = 101.5 ± 11.8, *p* < 0.0001), differences were absent at the 40th (*p* = 0.292) and 100th (*p* = 0.1198) percentiles (Table 1). The magnitude of LPS-induced change was consistently smaller than in 1G, and in some deciles the effect was negative, indicating a net reduction in MAPK activity after stimulation. This reflects limited inducibility under microgravity and points to altered signaling capacity.

Direct comparison of baseline conditions (1G vs. µG) revealed profound gravity-dependent effects. MAPK activity was significantly higher in µG across most deciles (e.g., 10%: 4447 vs. 2048, Δ = 2399 ± 324.5, *p* < 0.0001; 20%: 2538 vs. 1414, Δ = 1124 ± 67.2, *p* < 0.0001; 70%: 1024 vs. 858.0, Δ = 165.5 ± 88.4, *p* < 0.0001). Notably, at the 90th (*p* = 0.0611) and 100th (*p* = 0.2259) percentiles, these differences were not significant, indicating convergence of highly activated subpopulations irrespective of gravity. These data suggest that microgravity increases basal MAPK activation but limits the fraction of cells capable of reaching maximal activation (Table 1).

Comparison of LPS-treated samples (1G + LPS vs. µG + LPS) further supported this conclusion. Although significant differences persisted across most deciles (e.g., 20%: Δ = − 253.4 ± 51.3, *p* = 0.006; 60%: Δ = 160.4 ± 11.0, *p* < 0.0001; 80%: Δ = 99.9 ± 18.8, *p* < 0.0001), the magnitude of divergence was reduced compared to baseline, indicating that LPS stimulation partially normalizes the gravity-dependent differences.

Direct comparison of 1G + LPS and µg + LPS across deciles indicated no significant difference in the highest-activated subsets (90th and 100th percentiles), suggesting that cells already near maximal activation under µg do not further respond to LPS, consistent with a stress-induced ceiling effect.

At the 30th (*p* = 0.7577) and 100th (*p* = 0.4938) percentiles, no significant differences were detected, suggesting convergence of mid- and highly active subsets.

Taken together, these results demonstrate that LPS robustly activates MAPK under 1G but has minimal and inconsistent effects under µG, where microgravity elevates baseline activity and dampens responsiveness. The decile-ranking approach highlights increased population heterogeneity under µG, with greater variability and reduced inducibility across the distribution. These findings indicate that microgravity shifts immune cell signaling toward a dysregulated state characterized by elevated basal activity, blunted inducibility, and restricted maximal responsiveness patterns that would be obscured by bulk population averages.

### Fluorescence microscopy of monocyte activation

Fluorescence microscopy provided visual confirmation of functional analyses (Supplementary Fig. 1).

Cells were stained with an anti-pERK antibody (green) to visualize cytoplasmic MAPK activity and cell viability, and with DAPI (blue) to label cell nuclei. The green channel reflects cytoplasmic MAPK activity detected via the secondary antibody, whereas the blue channel (DAPI) identifies all cell nuclei, including those potentially lacking pERK immunoreactivity. In 1G + LPS cells displayed areas of concentrated green fluorescence, indicating high MAPK activity. Under µG fluorescence was heterogeneous, with scattered bright cells and some localized regions of concentrated signals.

µG + LPS showed some localized areas of concentrated fluorescence, but less pronounced than 1G + LPS, aligning with quantitative MAPK measurements and indicating reduced stimulus-induced responsiveness at the structural level. These observations are consistent with quantitative MAPK measurements and suggest that microgravity-induced cytoskeletal perturbations may contribute to uneven cellular activation.

These images reinforce that microgravity increases basal activity, reduces stimulus-induced responses, and enhances heterogeneity, while subsets of cells maintain high activation potential. The combination of global, single-cell, decile-ranked, and imaging analyses provides a comprehensive picture of how microgravity reshapes monocyte functional states.

## Discussion

Our study demonstrates that short-duration real microgravity (5 min aboard a sounding rocket) profoundly alters monocyte MAPK signaling, affecting basal activity, responsiveness to LPS, and population heterogeneity. Unlike previous studies that largely reported generalized suppression of immune signaling under microgravity (Okamura et al. 2024; Groot et al. 1990; Lv et al. 2023; Sonnenfeld 2002), our single-cell and population-level analyses reveal a more nuanced pattern: microgravity elevates basal MAPK activity in a subset of monocytes while simultaneously restricting the overall magnitude of LPS-induced responses. This combination of pre-activation and reduced inducibility has not been described in detail previously and may reflect a stress-induced shift in monocyte functional states.

It should be noted that the five-minute microgravity exposure in this study represents an acute model and may not fully recapitulate the chronic immunomodulatory effects observed during long-duration spaceflight. Nonetheless, early signaling changes, such as elevated basal MAPK activity and altered population heterogeneity, may represent initiating events that precede downstream immune adaptations. However, further studies should address the importance of the findings identified in the present study for long time microgravity exposure.

Elevated basal MAPK activity under microgravity suggests a compensatory or partially activated state (Verhaar et al. 2014). Mechanistically, altered cytoskeletal dynamics and disrupted mechanotransduction in low-gravity environments can lead to constitutive activation of stress-related pathways, including MAPK (Okamura et al. 2024; Lv et al. 2023). Similar trends have been reported for p38 and ERK in human immune cells under microgravity (Groot et al. 1990; Verhaar et al. 2014; Chen et al. 2017), but our data extend these observations by showing that basal activation is heterogeneous across the monocyte population, highlighting that only a fraction of cells adopt this pre-activated state.

Microgravity likely acts as a general cellular stressor, pre-activating subsets of monocytes and thereby limiting further stimulus-induced MAPK activation. This raises the possibility that µg could be used experimentally to probe stress-response mechanisms in immune cells or potentially as a modulator in countermeasure studies. However, the exact molecular pathways mediating this effect remain to be elucidated, and other signaling networks may contribute to the observed dampening of LPS responses. In this context it is important to note that the LPS concentration used is relatively high and this may obscure effects, as was for instance also noted when studying LPS-induced pinocytosis in this experimental system (Peppelenbosch et al. 1999). Hence future experimentation would benefit from a more broader range of LPS concentrations.

Importantly, this early activation pattern is consistent with reports in other immune lineages, including T cells, where rapid ERK responses are modified during short exposures to microgravity (Tauber et al. 2013). Broader analysis have shown, that ERK signaling is part of a wider network of gravity-sensitive pathways that adapt on both acute and longer timescales (An et al. 2024). Moreover long duration spaceflight studies have demonstrated that ERK activity contributes to macrophages differentiation and immune homeostasis (Shi et al. 2021), suggesting that the early signaling changes observed here may represent an initiating step in downstream immune remodeling.

LPS-induced MAPK responses were significantly blunted in microgravity-exposed monocytes. Critically, single-cell distributions indicate that while some cells can still reach high activation levels, the majority remain unresponsive. This suggests that microgravity not only dampens signaling amplitude but also disrupts coordination across the population. Existing literature describes general reductions in cytokine production and receptor-mediated signaling under microgravity (Ullrich et al. 2008; Wu et al. 2024; Ludtka et al. 2021), yet our study provides quantitative evidence at the single-cell level, demonstrating altered functional heterogeneity. This distinction is important because traditional bulk assays could underestimate the fraction of highly active or refractory cells, potentially masking key functional changes relevant for immune defense (Jew et al. 2020).

Fluorescence microscopy further corroborates these functional alterations (Wu et al. 2024; Garbacki et al. 2023). Under normal gravity, LPS stimulation promotes clear clustering of activated monocytes, whereas microgravity induces more scattered and heterogeneous patterns, consistent with our quantitative MAPK measurements (Li et al. 2018; Vroom et al. 2022). The combination of imaging and single-cell analyses allows us to link structural organization to signaling outcomes, supporting a model in which cytoskeletal perturbations in microgravity contribute to uneven MAPK activation and impaired population-level responsiveness.

A key technical aspect of this study relates to the limited number of analyzed samples (31 of 96 planned). This loss was primarily due to the highly constrained operational timeline during the sounding rocket campaign, including delayed automated fixation, incomplete fluid transfer in some hardware units, and mechanical stress during launch and landing. These factors led to compromised sample integrity in several cases, making them unsuitable for quantitative single-cell analysis. Importantly, all retained samples met predefined quality control criteria based on cell viability and staining intensity. Additionally, variation introduced by manual handling during the post-flight staining procedure may have influenced cell distribution and clustering patterns; however, all microgravity and 1G control samples were processed in parallel using identical protocols to minimize systematic bias. Nevertheless future studies addressing the effect of microgravity on monocyte clustering are called for. By explicitly documenting these technical limitations, our study aims to enhance transparency and reproducibility for future spaceflight immunology experiments.

We cannot exclude the possibility that microgravity-induced stress or selective loss of highly activated cells contributes to the observed reduced LPS inducibility. However, all analyzed samples met strict viability criteria (> 98%), and post-flight handling was standardized for all conditions. Thus, while minor contributions of stress-induced cell loss may occur, the heterogeneous responses likely reflect genuine signaling adaptation under microgravity rather than artifactually reduced responses.

Future studies could compare µg exposure to alternative stressors or employ clinostat-based simulated microgravity to differentiate specific gravity-dependent effects from general stress responses. Additionally, titration of LPS or alternative stimuli in partially activated cells may clarify whether µg limits maximal responsiveness or selectively affects subpopulations.

Our findings have direct implications for spaceflight immunology. The partial pre-activation and reduced inducibility may compromise coordinated inflammatory responses, increasing susceptibility to infection or dysregulated inflammation during prolonged low-gravity exposure. Furthermore, the observed heterogeneity suggests that therapeutic interventions aiming to restore immune function in space must account for variable cellular states rather than targeting average population responses.

In conclusion, our study provides the first comprehensive single-cell and population-level characterization of monocyte MAPK signaling under short-duration microgravity. While the 5-minute exposure represents an acute rather than chronic condition, it offers a physiologically relevant model of rapid immune signaling adaptation. By integrating quantitative, single-cell, and imaging data, we demonstrate that microgravity induces a complex remodeling of monocyte functional states, highlighting both elevated basal activity and restricted stimulus-induced responsiveness. These findings provide insight into the early events of immune dysregulation under microgravity, which may inform strategies to monitor or mitigate immune dysfunction during spaceflight and in terrestrial conditions involving stress-induced immune heterogeneity.

## Supplementary Information

Supplementary Material 1: Supplementary Figure 1. Fluorescenceimages of human monocytes under 1G and microgravity (µG) conditions, with andwithout LPS stimulation. Green: cytoplasmic MAPK activity/viability (anti-pERK antibody); Blue nuclei (DAPI). Localized regions of high fluorescence indicate activated cells; quantitative clustering was not measured. 1G + LPS shows areas of concentrated signal, while µG and µG + LPS show more scattered activation patterns, consistent with reduced stimulus responsiveness under microgravity.

Supplementary Material 2: Supplementary Table 1. Overview of donors included in the study.

## Data Availability

The data that support the findings of this study are available from the corresponding author upon reasonable request. The raw and processed datasets generated during the current study, including p44/42 MAPK activity measurements under different gravity and stimulation conditions, are not publicly available due to institutional data management policies and privacy restrictions.
