# Supplementary material for: Microgravity activates monocyte ERK1/2 signaling and modulates the response to lipopolysaccharide
Source: Mol Med. 2025 Nov 29;32:2. doi: 10.1186/s10020-025-01407-y (PMC12763956; doi:10.1186/s10020-025-01407-y)
Supplement: Supplementary file 1 — Supplementary Material 1: Supplementary Figure 1. Fluorescenceimages of human monocytes under 1G and microgravity (µG) conditions, with andwithout LPS stimulation. Green: cytoplasmic MAPK activity/viability (anti-pERK antibody); Blue nuclei (DAPI). Localized regions of high fluorescence indicate activated cells; quantitative clustering was not measured. 1G + LPS shows areas of concentrated signal, while µG and µG + LPS show more scattered activation patterns, consistent with reduced stimulus responsiveness under microgravity. [file 10020_2025_1407_MOESM1_ESM.docx]

**
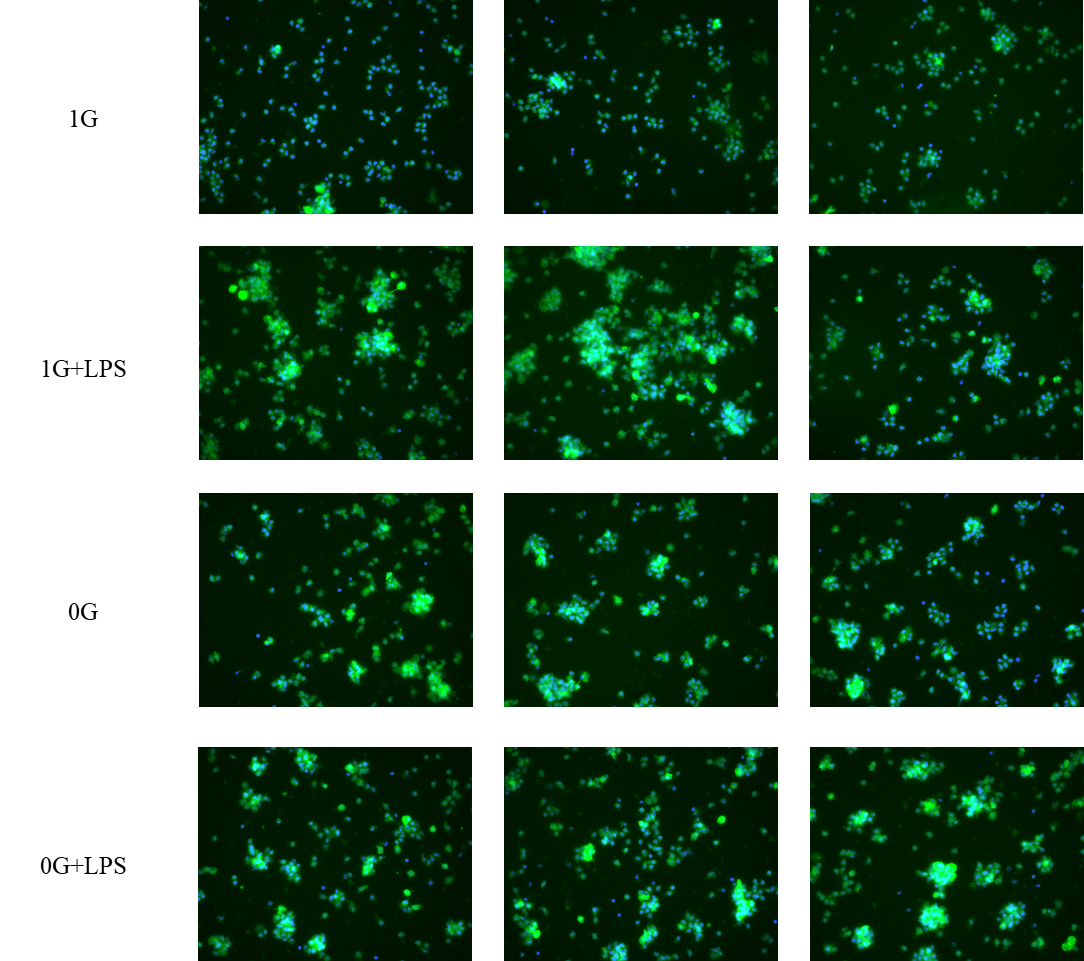
**

**Supplementary Figure 1. Fluorescence microscopy of monocytes under different conditions.**

4 × 3 grid shows experimental conditions (rows: 1G, 1G + LPS, 0G, 0G + LPS) across three donors (columns A–C). Green clusters indicate activation and aggregation. In 0G, fluorescence is heterogeneous; in 0G + LPS, clustering is less pronounced, reflecting reduced LPS responsiveness under simulated microgravity.
