# Supplementary material for: Microgravity activates monocyte ERK1/2 signaling and modulates the response to lipopolysaccharide
Source: Mol Med. 2025 Nov 29;32:2. doi: 10.1186/s10020-025-01407-y (PMC12763956; doi:10.1186/s10020-025-01407-y)
Supplement: Supplementary file 2 — Supplementary Material 2: Supplementary Table 1. Overview of donors included in the study. [file 10020_2025_1407_MOESM2_ESM.docx]

**Supplementary Table**

| **Mission** | **Year** | **Name** | **First name** | **Gender** | **Year of Birth** | **Blood used** |
| --- | --- | --- | --- | --- | --- | --- |
| Kappa | 2004 | K.K.Krisnadath | Shiela | ♀ | 1966 | Yes |
| Amuse | 2005 | Klaartje Kok | Klaartje | ♀ | 1975 | No |
|  |  | I.L.Huibregtse | Inge | ♀ | 1976 | No |
|  |  | S.H.Diks | Sander | ♂ | 1974 | No |
|  |  | M. Scheffer | Meike | ♀ | 1982 | No |
|  |  | S.H.Slofstra | Sjoukje | ♀ | 1977 | Yes |
| Micimmun | 2012 | E.Hoekstra | Elmer | ♂ | 1986 | Yes |
|  |  | W.K.Utomo | Wesley | ♂ | 1985 | No |
|  |  | S.W.A.Tjon | Angela | ♀ | 1983 | No |
|  |  | E.R.M.Bakker | Elvira | ♀ | 1982 | No |
|  |  | V.Dijkstra-Muncan | Vanesa | ♀ | 1974 | No |
|  |  | J.J.Deuring | Jasper | ♂ | 1985 | No |

**Supplementary table 1. Overview of donors included in the study.**
